# Sexually transmitted infections associated syndromes assisted in the primary health care in Northeast, Brazil

**DOI:** 10.1186/1471-2458-12-595

**Published:** 2012-08-02

**Authors:** Elani Graça Ferreira Cavalcante, Maria Alix Leite Araújo, Marli Teresinha Gimeniz Galvão, Heber José de Moura, Ana Paula Soares Gondim, Raimunda Magalhães da Silva

**Affiliations:** 1 Meireles Health Center, Av. Antonio Justa, 3113, Meireles, Fortaleza, Ceará, CEP 60.165-090, Brazil; 2 Av. Washington Soares, 1321, Edson Queiróz, Fortaleza, Ceará, CEP 60.811-905, Brazil; 3 Department of Nursing, Federal University of Ceará, Rua Alexandre Baraúna, 1145. Rodolfo Teófilo, Fortaleza, Ceará, CEP 60430-160, Brazil; 4 UNIFOR, Av. Washington Soares, 1321, Edson Queiróz, Fortaleza, Ceará, CEP 60.811-905, Brazil; 5 UNIFOR, Av. Washington Soares, 1321, Bairro Edson Queiróz, Fortaleza, Ceará, CEP 60.811-905, Brazil

**Keywords:** Sexually transmitted diseases, Primary health care, Aids serodiagnosis

## Abstract

**Background:**

The lack of information on the care for sexually transmitted infections (STI) associated syndromes may contribute for its non-inclusion as prevention and control strategy for STI in Brazil. This study aims to analyze the cases of STI – Associated Syndromes assisted in primary health care center in a city in Northeast Brazil associating them with socio-demographic and behavioral variables.

**Methods:**

This is a retrospective study that analyzed 5148 consultation forms and medical records of patients assisted in a primary health care center who presented at least one genital syndrome from 1999 to 2008. Was considered as dependent variables the genital syndromes and serologies for syphilis and HIV and as independent variables the socio-demographic and behavioral aspects. It was used Pearson’s chi-square test to analyze the differences between the categorical variables, with a significance level of 5%. It was performed a multivariate analysis through the multivariate logistic regression model with the variables with p <0.05. We used odds ratio with a confidence interval of 95%.

**Results:**

The most frequent syndromes were vaginal discharge and/or cervicitis (44%) and genital wart (42.2%). Most people were between 20 and 39 years old (70%) and women (74.2%). Genital ulcer was most prevalent among men (OR = 2.67; CI 95% 1.99-3.58) and people who studied more than eight years (OR = 1.33; CI 95% 1.00-1.75) and wart prevailed among men (OR = 3.92; IC 95% 3.36-4.57), people under 29 years old (OR = 1.81; CI 95% 1.59-2.07) and who studied more than eight years (OR = 1.75; CI 95% 1.54-1.99). The Venereal Disease Research Laboratory (VDRL) was positive in 7.3% of men and in 7.1% of women and the Anti-HIV in 3.1% of men and 0.7% of women.

**Conclusion:**

Vaginal discharge was the most frequent syndrome assisted in primary health care, followed by genital wart. The high prevalence of genital wart justifies the greater effort for the proper follow-up of these cases. Men presented more genital wart and ulcer and reported having more sexual partners, showing their need for a greater access and inclusion in health activities developed in primary health care in Brazil.

## Background

Prevention and control of sexually transmitted infections (STI) are challenges to be overcome all around the world and have gained importance again in public health context after evidences of their effects as cofactors for HIV transmission [1]. It is estimated that more than 448 million new cases of curable sexually transmitted infections (syphilis, gonorrhea, chlamydia and trichomoniasis) occur annually throughout the world [2], and if viral infections (human papillomaviruses, herpes simplex virus and HIV) were considered, this estimate would be substantially higher [3]. It is a problem that concerns the international community due to its serious implications for individual and collective health [3,4].

In Brazil, the epidemiological data on STI are scarce and the only ones which the notification is compulsory are Aids, HIV in pregnant women and exposed children, syphilis and urethral discharge syndrome in men [5]. Although the notification of these infections is compulsory, Brazilian health services do not perform it systematically, what causes an unawareness of the real magnitude of the problem and makes it difficult to develop strategies for prevention and control.

The Ministry of Health (MH) of Brazil, aiming to know the prevalence of these pathologies in general population (pregnant women and industry workers) and in vulnerable populations (people attending health clinics for care for STI), performed a multi-centered study in six Brazilian capitals. It identified prevalence of curable STI (syphilis, gonorrhea and chlamydia) in 13.5 % of pregnant women, 6.2 % of industry workers and 19.7 % of people attending health clinics for care for STI [6].

The data found in this study guarantee what is recommended by international institutions [7], i.e., that prevalence higher than 5% of curable STI imposes an enormous burden of morbidity in these capitals and probably in Brazil as well, reinforcing evidences that point out to failures in its prevention and control actions [6,8].

Concerning preventive actions, the predominant mode of transmission of STI is sexual, same as HIV, however, in some countries, there’s a great number of people and financial efforts for actions aimed at aids [9]. This can be noticed in Brazil through the efforts to reduce the vertical transmission of HIV when compared to the congenital syphilis [10]. On the other hand, epidemiological studies have acknowledged the treatment for STI as an important strategy to reduce the transmission of HIV, especially in countries where it is a recent epidemic [11].

Traditionally, STI were treated with an etiologic and clinical approach and both methods presented limitations. Then, there was the need to test other tools, what resulted in the development of the management of STI – Associated Syndromes through the use of flowcharts [12]. This strategy is recommended for places with few financial resources and personnel and with a poor access to laboratory tests.

The difficulties in the etiologic assessment of syndromes in Brazil led the Ministry of Health to recommend, from 1993, the management of STI – Associated Syndromes through the use of flowcharts. These ones were validated [13] and, due to limitations in the diagnosis of vaginal discharge syndrome [14], its flowchart has been reformulated in attempt to improve the positive predictive value. It also recommends that professionals assisting STI – Associated Syndromes shall make use of the Venereal Disease Research Laboratory test (VDRL), serology for HIV and most recently for Hepatitis B and C [14].

Studies on STI – Associated Syndromes in primary health care have not been performed in Brazil. There is a lack of information to help characterize the demand of people assisted in these services, and it makes it difficult to know the most prevalent syndromes and to identify the cases of syphilis and HIV.

The lack of visibility of the problem of STI and of the strategies adopted for its prevention and control may contribute for its non-inclusion in the primary health care system in Brazil. Generally, the health services are not properly prepared for this demand, maybe due to the lack of qualified professionals or organizational and operational conditions of these services.

Thus, this study aimed to analyze the cases of STI – Associated Syndromes assisted in a primary health care center in a city in Northeast Brazil, associating them with socio-demographic and behavioral variables and with the results of the VDRL and Anti-HIV tests.

## Methods

### Setting

We conducted a retrospective study that analyzed cases of STI – Associated Syndromes assisted in a primary health care center from 1999 to 2008. We considered for this study, the STI – Associated Syndromes established by the MH of Brazil (ulcer, discharges, warts and pelvic pain) [14].

The health care unit is part of an internship program where students from public and private universities of Fortaleza perform practical activities and it is also a place for research by national and international institutions. It holds an average of 120,428 consultations, and among these, 687 are consultations of people with genital complaints. It is, therefore, an ideal place for this research due to its care management and the availability of records on STI – Associate Syndromes.

The study took place in Fortaleza, capital of Ceará state, located in Northeast Brazil, where health care services are provided free of charge by the Brazilian National Health System (SUS). This system is a result of the struggle of social movements and was created after the Brazilian Constitution of 1988. One of its principles is to assure that health care is provided, free of charge, for everyone. In Fortaleza, this service is composed of 1116 health care units, 396 health care centers, 252 hospitals and also public and private laboratories and blood banks.

In the mid-90’s, a convention among the University of Bordeaux and the Ministry of Health and the Departments of Health Care Services of Ceará and Fortaleza was signed for the implementation of care services for STI – Associated Syndromes through the syndrome management. This convention enabled the continuous education and training of professionals with emphasis on reception and counseling. It also enabled the acquisition of medicines, material and the internal patient flow management. Since then, the health care unit keeps all the resources available, especially medicines and the technical support for the collection of serologies for syphilis and HIV after consultations.

From 1999 to 2008, the unit assisted 6872 people with genital syndromes. We analyzed the cases that presented only one STI – Associated Syndrome (ulcer, discharges, warts, pelvic pain). Then, 1724 people who presented more than one genital syndrome were excluded. The remaining 5148 cases were analyzed in this study.

The data collection was done through the specific forms for services for STI and patients’ records. This form was implemented in the unit in 1999 and since then it is filled and typed everyday by a professional who is specifically in charge of it. The data that were not in the forms and records were classified as ignored. The cases of Hepatitis B and C were not analyzed because such tests were not performed in the unit during the period that it was studied.

The returns of these patients for reassessment are scheduled according to the time the results of the VDRL and Anti-HIV tests come out (seven days for VDRL and thirty days for Anti-HIV). In this study, we considered that the patient returned to the unit for reassessment within three months from the first consultation.

### Biological specimens and serology

The syndrome management of STI – Associated Syndromes aims to provide in a single consultation, the diagnosis, treatment and counseling in order to immediately break the transmission chain effectively [14,15]. In Brazil, all the flowcharts recommend as a complementary action the serology tests VDRL, HIV and Hepatitis B and C along with counseling, as well as the notification and treatment of sexual partners.

The quantitative and qualitative VDRL tests are collected and done in the unit. We considered reactive for syphilis all patients with reactive VDRL equals to or higher than 1:2, since they haven’t presented previous treatment. The VDRL is an available test that is largely used in primary health care in Brazil, mainly due to its simplicity and low-cost. It’s worth saying that Brazil aims to control congenital syphilis and all efforts have been made to identify suspected cases of syphilis, which justifies the serological diagnosis through non-treponemic tests and the treatment of reactive cases in places where confirmatory tests are unavailable [16].

The serological sample for Anti-HIV is collected in the health center after informed consent is obtained during counseling. After collection, the material is sent to the state of Ceará reference laboratory (Central Laboratory of Ceará – LACEN), responsible for most of the serological diagnoses of HIV infections in the State and that has always met the technical rules standardized in the MH of Brazil [17]. This diagnosis is performed in two stages: a classification through the Enzyme Linked Immunoabsorbent Assay (ELISA) technique and, regarding the result, a complementary with the Indirect Immunofluorescence (IFI) technique. The medical report comes out when the results are positive in stages I and II.

Brazilian rules require the collection of a second blood sample to prove laboratorial diagnoses of positive cases. The collection must be performed during the delivery of the first reactive sample. It’s worth saying that the second sample undergoes only through stage I of the flowchart. In this study, the reactive cases were the ones which the results were positives in the first and second sample.

Non-reactive results in the first stage are released and taken to the health care center to be delivered to patients, with a post-test counseling when a second blood sample collection is required after 30 days, in case the patient is in the immunological window period.

### Statistical analysis

We considered as dependent variables the types of syndromes and the VDRL and Anti-HIV tests because they were part of the daily health care of patients with STI – Associated Syndromes. The independent variables were city of residence, sex, age (years), schooling (studying years), return for reavaliation and number of sexual partners in the past three months, once these information were in the standardized consultation forms.

The data were typed in the statistical package *Epi-Info 6.04* (Centers for Disease Control and Prevention – CDC - Atlanta, Georgia, USA) and analyzed in the software *SPSS 19.0* (IBM Company, Chicago, USA). We used Pearson’s Chi-square test to analyze the differences among the categorical variables, with a significance level of 5%. A multivariate analysis was performed by the statistical package *STATA 11.0* (Stata Corp LP, College Station, TX 77845, USA), through a logistic regression model, using the stepwise technique. For the adjusted analysis we considered the variables with p values p <0.05. We used the Odds Ratio (OR) with a 95% confidence interval as an effect size. The syndromes assessed in the logistic regression were warts and genital ulcers, since they occur in both men and women.

The study as approved by the Ethics in Research Committee of the Public Health School of Ceará according to Protocol n^o^ 126/2008.

## Results

We analyzed 5148 forms of people with at least one STI – Associated Syndrome (74.2% women and 25.8% men). Table 1 shows the socio-demographic variables. Most of the people were in the age group between 20 and 29 years old (46.9%) and 16.2% were under 20. Regarding schooling, 64.0% of patients (average 7, SD 3.4) have studied for less than eight years. Regarding the number of sexual partners in the past three months, 79.2% reported having only one partner and among these, 80.1% were women. Among the 672 (12.4%) patients who reported having two or more sexual partners, 422 (67.3%) were men. Among children under 12 years old, five (45.4%) reported having sexual partners and all these were between 11 and 12 years old.

**Table 1:** Socio-demographic, behavioral, VDRL and anti-HIV tests variables in assisted with STI-Associated Syndromes in primary health care, Fortaleza, Ceará, Brasil, 1999 – 2008

**Variables**	**n**	**%**
City of residence		
Fortaleza	5010	97.3
Other	138	2.7
Sex		
Male	1326	25.8
Female	3822	74.2
Age (years)		
≤ 19	831	16.2
20 – 29	2416	46.9
30 – 39	1191	23.1
≥ 40	710	13.8
Schooling (studingyears)		
0 - 4	1337	26.0
5 – 8	1955	38.0
≥ 9	1813	35.2
Unknown	43	0.8
Number of sexual partners (last three months)		
None	358	7.0
1	4076	79.2
> 1	627	12.1
Unknown	87	1.7
Return for reavaliation		
Yes	4631	90.0
No	517	10.0
Total	5148	100.0

**Figure 1:**
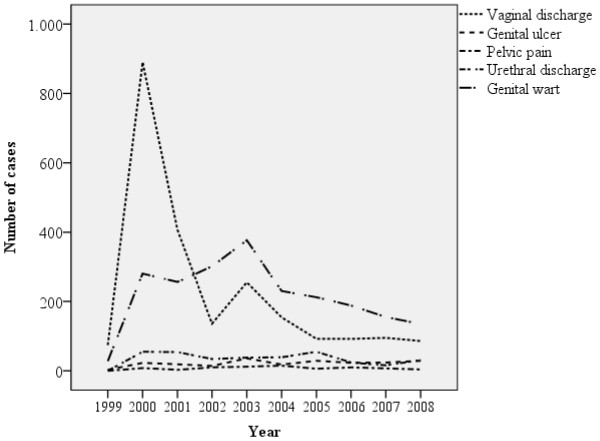
**STI-Associated Syndromes assisted over the years in primary in health care.** Fortaleza, Ceará, Brazil. 1999 a 2008.

The most frequent syndrome was vaginal discharge and/or cervicitis (44.5%), followed by genital wart (42.2%), urethral discharge (5.2%), genital ulcer (4.8%) and pelvic pain (1.4%). Figure 1 shows the annual distribution of STI – Associated Syndromes according to the consultations in the unit from 1999 to 2008. In the first three years the most prevalent cases were vaginal discharge and/or cervicitis, which have decreased along the years. It is possible to notice that the number of people with genital wart who attended the unit stands out among the other syndromes and it doesn’t change along the years.

Table 2 shows an analysis of the socio-demographic variables and the syndromes that occur in both men and women (genital ulcer and wart). The rate of genital ulcer and wart was higher in men (9.9% and 63.9%), among people who studied more than eight years (6.2% and 53.4%) and people who reported having two or more sexual partners in the past three years (8.9% and 45.6%). The rate of cases was higher in people who were 29 years old or younger only for genital wart (53.4%).

**Table 2:** Multivariate logistic regression analyses for genital ulcer and warts according to sex, age, schooling and number of sexual partner in primary health care center, Fortaleza, Ceará, Brazil, 1999-2008

**Variables**	**Genital Ulcer**								**Genital Warts**							
			**Not adjusted**			**Adjusted**					**Not adjusted**			**Adjusted**		
	n/N*	**%**	**OR**	**IC (95%)**	**p value**	**OR**	**IC (95%)**	**p value**	n/N*	**%**	**OR**	**IC (95%)**	**p value**	**OR**	**IC (95%)**	**p value**
Sex																
Male	131/1326	9.9	3.11	2.40-4.03	<0.001	2.67	1.99-3.58	<0.001	848/1326	63.9	3.33	2.92-3.81	<0.001	3.92	3.36-4.57	<0.001
Female	130/3822	3.4	1						1326/3822	34.7						
Age (years)																
≤ 29	157/3090	4.84	1						1579/3247	48.6	2.07	1.84-2.34	<0.001	1.81	1.59-2.07	<0.001
> 29	104/1901	5.47	1.14	0.87-1.48	0.315	1.24	0.93-1.64	0.135	595/1901	31.3	1					
Schooling (studingyears)																
≤ 8	148/3292	4.5	1						1188/3292	36.1	1					
> 8	112/1813	6.2	1.39	1.07-1.81	0.008	1.33	1.00-1.75	0.044	969/1813	53.4	2.03	1.80-2.28	<0.001	1.75	1.54-1.99	<0.001
N^o^ partners (last three months)																
1	174/4076	4.3	1						1699/4076	41.7	1					
≥ 2	56/627	8.9	2.19	1.57-3.03	<0.001	1.17	0.98-1.39	0.071	286/627	45.6	1.17	0.98-1.39	0.063	0.54	0.44-0.66	<0.001

*n/N = number of VDRL tests reagent results and the number of sample tested.

**n/N = number of HIV tests reagent results and the number of sample tested.

The multiple regression analysis showed a significant association between genital ulcer and the variables sex (OR = 2.67; CI 95% 1.99-3.58) and schooling (OR = 1.33; CI 95% 1.00-1.75), and between genital wart and sex (OR = 3.92; CI 95% 3.36-4.57), age (OR = 1.81; CI 95% 1.59-2.07) and schooling (OR = 1.75; CI 95% 1.54-1.99) (Table 2).

In general, the positivity for VDRL and HIV was 8.2% and 1.3% respectively. The result for VDRL was reactive in 7.3% of men and 7.1% of women, and for Anti-HIV was 3.1% for men and 0.7% for women. There were no reactive cases of VDRL and Anti-HIV in children under 12 years old. The multiple regression analysis showed a significant association between schooling and the reactive result of VDRL (OR = 1.87; CI 95% 1.44-2.43). There was no significant association between reactive cases of HIV and the variables analyzed (Table 3).

**Table 3:** Multivariate logistic regression analyses for VDRL and anti-HIV test reagents according according to sex, age, schooling and number of sexual partner in primary health care center, Fortaleza, Ceará, Brazil, 1999-2008

**Variables**	**VDRL reagent**								**HIV reagent**							
			**Not adjusted**			**Adjusted**					**Not adjusted**			**Adjusted**		
	n/N*	**%**	**OR**	**IC (95%)**	**p value**	**OR**	**IC (95%)**	**p value**	n/N*	**%**	**OR**	**IC (95%)**	**p value**	**OR**	**IC (95%)**	**p value**
Sex																
Male	97/1326	7.3	1						30/952	3.1	1					
Female	271/3822	7.1	1.03	0.80-1.32	0.784	0.93	0.71-1.22	0.642	22/3110	0.7	0.25	0.14-0.43	<0.001	0.35	0.18-0.64	<0.001
Age (in years)																
≤ 29	222/3247	6.8	1.13	0.90-1.40	0.257	0.98	0.78-1.24	0.925	31/3247	0.95	1.15	0.63-2.08	0.603	1.17	0.62-2.21	0.620
> 29	146/1901	7.7	1						21/1901	1.10	1					
Schooling (studingyears)																
≤ 8	276/3292	8.3	1						34/3292	1.03	1					
> 8	87/1813	4.8	1.81	1.41-2.35	< 0.001	1.87	1.44-2.43	<0.001	18/1813	0.99	1.04	0.56-1.96	0.891	1.05	0.55-1.98	0.880
N^o^ partners (last three months)																
1	293/4076	7,2	1.11	0.80-1.53	0.481	1.14	0.81-1.66	0.439	32/4076	0.79	2.25	1.02-4.62	0.017	1.22	0.58-2.57	0.594
≥ 2	50/627	8.0	1			1			11/627	1.15	1					

*n/N = number of VDRL tests reagent results and the number of sample tested.

**n/N = number of HIV tests reagent results and the number of sample tested.

## Discussion

It is important to add that the unit where this study took place was the first one to include the health care services for STI – Associated Syndromes in Fortaleza, which means that it required organization, a training program with all the professionals for the proper management of cases of STI, reception and counseling, and also the availability of inputs and medicines, which might have contributed for a better performance of the health team during the assistance of cases.

It was noticed that most people who looked for care were women, people within their reproductive age and with a low schooling. In Brazil, despite the efforts to improve actions for women’s health, the primary health care services are still directed to infant-maternal care [18]. In Fortaleza, the care for STI was implemented in 1999 and most of the people who looked for care were women, which may be the reason for a large number of cases of vaginal discharge syndrome in the first three years after its implementation.

A low percentage of men looked for care in this unit. This low demand may be related to men’s difficulty to access health care services [19], sociocultural and gender peculiarities [18,20] and the difficulties for health professionals to properly manage cases, once they represent barriers to be overcome in order to effectively control STI in Brazil. Actions directed to men’s health are still being implemented in the country [21].

It is important to highlight the presence of children among the people who looked for care and its implications when it derives from sexual abuse [22]. In Brazil, despite the policies aimed at prevention and treatment of harms resulting from sexual abuse [23], there are many barriers for the management of these cases such as the victims themselves and their families, the professionals and mainly the lack of an organized care system for this demand.

There was a higher rate of vaginal discharge syndrome and/or cervicitis among the cases. Although vaginal discharge is not necessarily related to a STI, it is one of the most frequent reasons for looking for care in of sexually transmitted diseases (STD) services in Brazil [6] and evidences show that it can increase the risk to acquire HIV [24,25], what reinforces the importance of the inclusion of its management in primary health care. In Brazil, this flowchart was validated and improved in attempt to increase its predictive value in the diagnosis of cervicovaginal diseases [13].

The analysis of the syndromes that occur in both men and women showed that genital wart and ulcer were most prevalent among men, a result that is similar to the one found in a study performed in Brazil [6]. Considering this and the fact that ulcers have been found to be associated with increased risk of HIV transmission and genital warts to be powerful predictors for Human Papillomavirus (HPV) [6], there is an important need for treatment and/or follow-up of people, especially men, in primary health care. However, there are many draw-backs for the management of cases in health services due to operational conditions, lack of training programs and professionals’ resistance to perform the treatment.

Although most of the people have reported having only one sexual partner in the past three months, the genital wart and ulcer syndromes were most prevalent in people with two or more partners. It’s worth saying that, although most of the people were women, men were the ones who reported having more sexual partners, confirming the findings of other studies in Brazil which report that the exchange of sexual partners is more common among men [26]. Despite the possibility of information bias, which certainly limited the analysis of this variable, we consider that this finding can represent the demand of people who look for care in the primary health care in Brazil, mainly women who do not run an imminent risk of STI and HIV but are vulnerable to them [26,27].

We found a high prevalence of reactive VDRL (8.2%), higher than the one found in a study conducted with people who looked for care in STD clinics in Brazil [6]. This was probably due to the unavailability of confirmatory tests for the diagnosis of syphilis in the primary health care. Moreover, the VDRL test is considered a powerful predictor of syphilis, even in cases with low titration [28], and its use in public health is internationally recommended for places where the diagnosis confirmation is not available. For this reason, it is recommended that all people with reactive VDRL shall be treated for syphilis [3].

The identification of 1.3% of reactive serology for HIV among people with STI – Associated Syndromes calls the attention to the importance of tests for people with genital syndromes. In Brazil, the access to anti-HIV testing is provided only for pregnant or parturient women and for people who attend Testing and Counseling Centers and blood banks [29].

It was observed a great use of VDRL and HIV tests, which might have happened due to the unit management system. The unit collects blood samples right after the consultation, with a pre and post-test counseling. The high percentage of people who return to the unit points out to a good acceptance of its system and can be considered an important indicator of the good quality of its services.

However, it’s worth saying that the lack of records of confirmatory tests for syphilis, data on notification of partners and also on patients’ sexual orientation has limited the analysis of this study because it hindered the gathering of further information on the people assisted and their association with the variables analyzed.

It is important to say that the care for STI – Associated Syndromes in primary health care requires motivated and trained personnel, availability of medicines, accessible and acceptable health services and also an improvement in quality of records. Such records could provide information for a better understanding of the demand of people with STI – Associated Syndromes assisted in the unit and contribute to the development of feasible prevention strategies.

## Conclusion

This study enabled to identify that vaginal discharge was the main STI – Associated Syndrome assisted in primary health care followed by genital wart. The high prevalence of genital wart justifies the implementation of efforts for a proper follow-up of these cases. Men presented more wart and genital ulcer syndromes and reported having more sexual partners, calling the attention to the need to improve their access and inclusion in health activities developed in the primary health care in Brazil.

## Competing interests

We recognize that this manuscript does not present financial conflicts among the authors. We certify that this manuscript was submitted only and exclusivity to the BMC Public Health.

## Authors’ contributions

EGFC developed the project, collected, analyzed and interpreted data. Maria Alix Leite Araujo – developed the project, analyzed and interpreted data, and helped in the final review of the article. MTGG developed the project, helped in the draft and final review of the article. HJM performed the statistical treatment, analyzed data and helped in the final review of the article. APSG analized and interpreted data and helped in the final review of the article. RMS analyzed and interpreted data and helped in the final review of the article. All authors read and approved the final manuscript.

## Authors’ information

Elani Graça Ferreira Cavalcante, MS, Secretary of the State of Ceará.

Maria Alix Leite Araujo, PhD, Professor, University of Fortaleza.

Marli Teresinha Gimeniz Galvão, PhD, Professor, Federal University of Ceará.

Heber José de Moura, PhD, Professor, University of Fortaleza.

Ana Paula Soares Gondim, PhD, Professor, University of Fortaleza.

Raimunda Magalhães da Silva, PhD, Professor, University of Fortaleza.

## Pre-publication history

The pre-publication history for this paper can be accessed here:

http://www.biomedcentral.com/1471-2458/12/595/prepub
